# Altered fecal microbiome and metabolome profiles in rat models of short bowel syndrome

**DOI:** 10.3389/fmicb.2023.1185463

**Published:** 2023-06-09

**Authors:** Yuhua Huang, Jian Jiao, Danhua Yao, Feilong Guo, Yousheng Li

**Affiliations:** ^1^Department of General Surgery, Shanghai Ninth People’s Hospital, Shanghai JiaoTong University School of Medicine, Shanghai, China; ^2^Department of General Surgery, Jinling Hospital, School of Medicine, Nanjing University, Nanjing, China

**Keywords:** short bowel syndrome, intestinal failure, microbiome, metabolome, intestinal adaptation

## Abstract

**Introduction:**

Short bowel syndrome (SBS) is featured by impaired nutrients and fluids absorption due to massive small intestine resection. Gut dysbiosis has been implicated in SBS, this study aimed to characterize the metagenomic and metabolomic profiles of SBS and identify potential therapeutic targets.

**Methods:**

Fecal samples from SBS and Sham rats (*n* = 8 per group) were collected for high-throughput metagenomic sequencing. Fecal metabolomics was measured by untargeted liquid chromatography-mass spectrometry.

**Results:**

We found that the species-level α-diversity significantly decreased in SBS rats, accompanied by altered microbiome compositions. The beneficial anaerobes from Firmicutes and Bacteroidetes were depleted while microorganisms from *Lactobacillus*, *Escherichia*, *Enterococcus*, and *Streptococcus* were enriched in faces from SBS rats. LEfSe analysis identified 17 microbial species and 38 KEGG modules that were remarkably distinct between SBS and Sham rats. In total, 1,577 metabolites with known chemical identity were detected from all samples, among them, 276 metabolites were down-regulated and 224 metabolites were up-regulated in SBS group. The typical signatures of SBS fecal metabolome comprised reduced short-chain fatty acids and products of amino acid metabolism (indole derivatives and p-cresol), as well as altered bile acid spectrum. We revealed 215 robust associations between representative differentially abundant microbial species and metabolites, the species with the same changing trend tended to have a similar correlation with some certain metabolites.

**Conclusion:**

The fecal microbiome and metabolome significantly altered in SBS. Our findings may lay the foundation for developing new strategies to facilitate intestinal adaptation in SBS patients.

## Introduction

Extensive loss of functional small intestine due to surgical resection may result in short bowel syndrome (SBS), which is a most common type of intestinal failure (Pironi, 2016). In SBS, inadequate intestinal surface area causes impaired absorption of nutrients and fluids, thus, patients need to depend on sustained parenteral nutrition (PN) to maintain healthy and/or growth (Neelis et al., 2019). However, adaptative process of the intestine which includes bowel lengthening and thickening, increases in villus height and crypt depth, increased nutrient transporter expression, accelerated crypt cell proliferation, and slowed transit time, may occur in patients with SBS, especially during the first 2 years after surgery, to compensate for the malabsorption (Tappenden, 2014). Sufficient intestinal adaptation may ultimately allow patients with SBS to wean off PN and have good clinical outcomes (Jeppesen, 2014). Remarkably, gut microbiota has been identified as a determinant of the post-resection adaptation in SBS (Gillard et al., 2017).

Alterations of the gut environment, including lower luminal pH, elevated luminal oxygen concentration, disrupted enterohepatic circulation of bile acids (BAs) and indigestive substrates, lead to significant intestinal dysbiosis in SBS (Budinska et al., 2020). Prior studies have reported decreased diversity and richness of gut microbiota in pediatric and adult SBS patients (Engstrand Lilja et al., 2015; Huang et al., 2017). Moreover, the SBS patients commonly exhibited an overabundance of Proteobacteria and its family *Enterobacteriaceae*, the pathogens within which could cause chronic intestinal inflammation and even liver disease by producing lipopolysaccharide (Korpela et al., 2017). Besides, a higher abundance of Firmicutes and a large decrease of Bacteroidetes have also been reported (Davidovics et al., 2016; Budinska et al., 2020; Boutte et al., 2022; Fourati et al., 2023). Additionally, although the commensal genera from Firmicutes and Bacteroidetes were usually depleted (Huang et al., 2017; Piper et al., 2017; Wang et al., 2017; Engelstad et al., 2018), some beneficial microbes belonging to *Lactobacillaceae* could be dominant in SBS individuals (Joly et al., 2010; Engstrand Lilja et al., 2015; Fourati et al., 2023). However, most studies relied on the 16S ribosomal RNA (rRNA) gene sequencing method, which lacks enough sequencing depth and cannot provide quantitative functional annotation. The metagenomic sequencing may offer deeper insights into the compositional and functional changes of gut microbiome in SBS (Piper et al., 2017).

In addition to the direct effects of shifted microbial profiles, the metabolites, either derived from bacterial fermentation of dietary substrates or produced directly by gut microbes, also contribute significantly to the host physiological functions. For example, the short chain fatty acids (SCFAs), a production of specific gut microbes via fermentation of non-digestible carbohydrate, have been shown to nourish intestinal epithelial cells, modulate immune response and improve intestinal adaptation in SBS (Kles and Chang, 2006; Marchix et al., 2018). Recently, the association between gut microbiome and BAs metabolism in SBS patients has also attracted growing attention (Kastl et al., 2022). Fecal metabolomics is considered as the functional readout of the gut microbiome (Zierer et al., 2018), yet, the characteristics of fecal metabolome in SBS are still unclear.

Hence, in present study, we performed high-throughput metagenomic sequencing and untargeted liquid chromatography-mass spectrometry (LC-MS) to analysis the fecal microbiome and metabolome profiles of rat SBS models. Based on these combined strategies, we aimed to present comprehensive characterization of gut microbes and metabolites specific to the particular luminal conditions of SBS individuals.

## Materials and methods

### SBS rat model and fecal sample collection

Four-week-old male Sprague–Dawley (SD) rats were purchased from Caves Laboratory Animal Co., Ltd. (Changzhou, China) and housed in groups of four in an animal facility with a 12-h light-dark cycle. Animals were allowed to access water and solid chow *ad libitum* during a 1-week acclimatization period. At 5 weeks of age, all rats were kept on a nutrient-fortified water gel (DietGel Recovery, ClearH_2_O, Westbrook, ME, USA) for another 1 week.

At 6 weeks of age, rats were randomly assigned to Sham or SBS group (8 rats each group) and anesthetized by inhalation with 2% isoflurane before surgery. Rats in SBS group underwent a resection of 75% of the small intestine, including the ileum along with the ileocecal junction, i.e., 75 cm proximal to the ileocecal junction and 1 cm distal to the cecum, followed by jejuno-colonic anastomosis. The sham operation was performed by transection and re-anastomosis of the small bowel approximately 75 cm proximal to the ileocecal junction. The resection margins were anastomosed by an end-to-end, single-layer method using 7–0 braid sutures. The rats were resuscitated by intraperitoneal injection of 4 ml 0.9% saline solution and kept in a warm incubator with free access to water for the first postoperative 24 h. After that, rats were housed individually, fed on the nutritionally fortified water gel (DietGel Recovery, ClearH_2_O, Westbrook, ME, USA) for 1 week and then allowed water and solid chow *ad libitum* until the end of the study.

On postoperative day 28, the fecal sample of each rat was collected using sterile cryopreservation tubes, flash frozen in liquid nitrogen and stored at −80°C until analysis. This study was approved by the Animal Experimental Ethics Committee of the Shanghai Ninth People’s Hospital, Shanghai Jiao Tong University School of Medicine (Approval No. SH9H-2019-A174-1).

### Metagenomic analysis of fecal samples

Total genomic DNA was extracted from approximately 200 mg of fecal sample using E.Z.N.A. Soil DNA Kit (Omega Bio-tek, Norcross, GA, USA) in accordance with the manufacturer’s instructions. The DNA concentration was measured by Quantus Fluorometer using the PicoGreen assay (Promega, Madison, WI, USA). The purity (OD 260/280) and integrity of the extracted DNA were determined using NanoDrop 2000 spectrophotometer (Thermo Fisher Scientific, Wilmington, DE, USA) and agarose gel electrophoresis, respectively. After the fragmentation of DNA to an average size of about 400 base pairs (bp), the paired-end library was generated using NEXTflex Rapid DNA-Seq kit (Bioo Scientific, Austin, TX, USA). The libraries for metagenomic analysis were sequenced on an Illumina NovaSeq 6000 platform (Illumina Inc., San Diego, CA, USA) with a targeted data size of 6.0 Gb per sample.

The raw sequences were quality filtered using Fastp (Chen et al., 2018) to trim adapter and remove low-quality reads (length < 50 bp or quality score < 20 or having ambiguous N bases), the remaining reads were next aligned to rat genome by the Burrows-Wheeler Aligner (Li and Durbin, 2009) to eliminate host DNA. *De novo* assembly of the clean reads were performed using MEGAHIT (Li et al., 2016) and only contigs with the length ≥300 bp were selected for further analysis. Open reading frames (ORFs) within assembled contigs were predicted using MetaGene (Noguchi et al., 2006), and all predicted genes were clustered by CD-HIT software (Fu et al., 2012) with a threshold of more than 95% sequence identify and 90% length coverage to construct a non-redundant gene catalog. The high-quality reads of each sample were then mapped to the non-redundant gene catalog with 95% identify using SOAPaligner (Li et al., 2008).

Taxonomic assignment of the predicted genes was conducted on the basis of NCBI-NR database using DIAMOND (Buchfink et al., 2015) with *e*-value ≤ 10^–5^. Subsequently, the predicted genes were compared with the Kyoto Encyclopedia of Genes and Genomes (KEGG) database using DIAMOND to obtain KEGG annotation and metabolic pathway information, also with *e*-value ≤ 10^–5^. α-diversity (within-sample diversity) including Chao1 and Shannon indices was calculated using the vegan package in R. Principal coordinates analysis (PCoA) was used to visualize β-diversity (between-sample diversity) based on Bray-Curtis distance, and permutational multivariate analysis of variance (PERMANOVA) was used to evaluate differences across groups. The significantly differential bacterial species or KEGG modules between groups were identified by Linear discriminant analysis (LDA) Effect Size (LEfSe) with a LDA score > 3.5 or 2.5, respectively.

### Untargeted LC-MS analysis

Each 50 mg fecal sample was weighted and mixed with 400 μl extract solution (methanol: water = 1:1 (v/v), containing 0.02 mg/mL L-2-chlorophenylalanin as internal standard). The mixture was allowed to settle at −10°C and homogenized at 50 Hz for 6 min, subsequently, it was ultrasonicated at 40 kHz for 30 min at 5°C. After incubation at −20°C for 30 min to precipitate proteins, the mixture solution was centrifuged at 13,000 *g* and 4°C for 15 min, and the resulting supernatant was collected for LC-MS analysis. The quality control (QC) sample was prepared by mixing an equal aliquot supernatant from each sample.

The LC-MS analysis was performed using an UHPLC system (Vanquish, Thermo Fisher Scientific, Wilmington, DE, USA) with an ACQUILY UPLC HSS T3 column (100 mm × 2.1 mm i.d., 1.8 μm; Waters, Milford, MA, USA) coupled to the Q Exactive HF-X mass spectrometer (Orbitrap MS, Thermo Fisher Scientific, Wilmington, DE, USA). The mobile phases consisted of 0.1% formic acid in water: acetonitrile (95:5, v/v) (solvent A) and 0.1% formic acid in water: acetonitrile: isopropanol (5:47.5:47.5,v/v) (solvent B). The sample injection volume was 2 μL with the flow rate set to 0.4 mL/min, and the column temperature was maintained at 40°C. The Thermo Q Exactive HF-X mass spectrometer was equipped with an electrospray ionization (ESI) source operating in either positive or negative ion mode to collect the mass spectrometric data. The heater and capillary temperatures were 425 and 325°C, respectively. The detection was carried out over a mass range of 70–1,050 m/z. The flow rates of sheath and aux gas were 50 and 13 arb, respectively. The normalized collision energy was set at 20/40/60 eV. The full MS resolution was 60,000 and MS/MS resolution was 7,500. Both the ESI + and ESI–ion-spray voltages were 3.5 kV. Data acquisition was performed with the Data Dependent Acquisition (DDA) mode.

Raw data of LC-MS were preprocessed by Progenesis QI software (Waters, Milford, MA, USA) for peak detection, filtering, alignment, and integration, and a dada matrix consisting of retention time, mass-to-charge ratio (m/z) values and peak intensity was exported. Each retained peak was normalized by sum normalization method. Variables with the relative standard deviation (RSD) > 30% of QC samples were removed. The Human Metabolome Database (HMDB) and the METLIN database were applied for metabolite annotation. The mass tolerance between the measured and theoretical masses of the components of interest was 10 ppm.

The detected metabolites were normalized and clustered by heatmap. Orthogonal partial least squares discriminant analysis (OPLS-DA) was performed to determine the differential metabolic profiles between Sham and SBS groups, and calculate the Variable importance for the projection (VIP) of metabolites. To avoid the risk of overfitting, seven-fold cross-validation was performed during modeling and validated by 200 response permutation testing. Student’s *t*-test was used to calculate *p*-value and fold change (FC) was obtained by univariate analysis. If VIP > 1 and *P* < 0.05, the variable was defined as a significantly different metabolite between two groups. The altered metabolites were mapped to their biochemical pathways through metabolic enrichment and pathway analyses according to a database search (KEGG).

### Statistical analysis

Bacterial relative abundance and α-diversity were compared via Mann–Whitney test using GraphPad Prism version 9.1.0 (Graphpad software, San Diego, CA, USA). Correlations between differentially enriched species (identified by LEfSe with LDA > 3.5 and *P* < 0.05) and metabolites (OPLS-DA VIP > 2 and *P* < 0.05) were evaluated by Spearman’s rank correlation analysis using the scipy package in python. Unless otherwise stated, *P* < 0.05 was considered as statistically significant.

## Results

### Survival and weight change of rats

Both the SBS and Sham groups had a 100% survival rate at postoperative day 28. The Sham rats had a weight loss at the first two postoperative days and naturally gained weight throughout the follow-up (Supplementary Figure 1A). On the contrary, the weight of SBS rats continued to drop during the first postoperative week and the lowest weight was observed at day 7 with a mean of 79.64 ± 4.31% of their initial weight (Supplementary Figure 1A). From day 8, weight of SBS rats increased slowly until the end of the follow up and remained significantly lower than that of Sham rats at day 28 (99.39 ± 8.53% vs. 125.65 ± 4.03%, *p* = 0.0002, Supplementary Figures 1A, B). Totally, 3 SBS rats recovered their original weight at postoperative day 28.

### Altered microbial community structure in SBS

**FIGURE 1:**
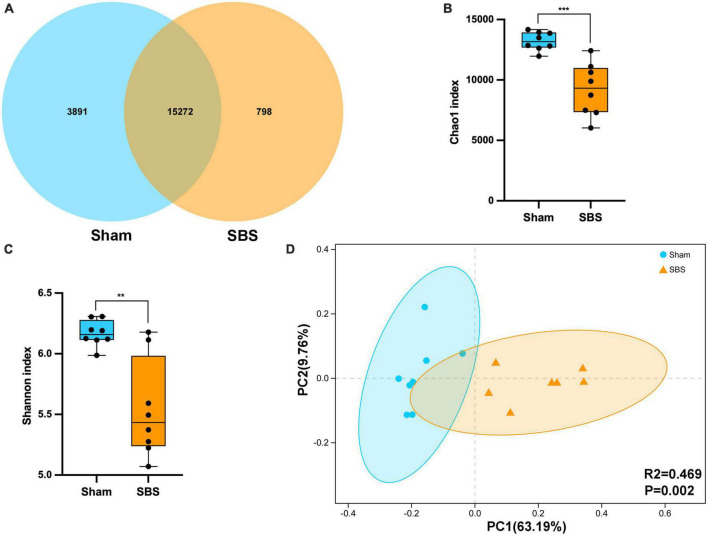
Fecal microbiome structure in short bowel syndrome (SBS) and Sham rats. **(A)** Venn diagram of microbial species in SBS and Sham groups. **(B,C)** Comparison of Chao1 **(B)** and Shannon **(C)** indices (α-diversity) of microbial communities at species level between SBS and Sham groups. The dot represents one value from individual participants. The box plots represent median and interquartile range (IQR), and the whiskers indicate the 10th and 90th percentiles. **p < 0.01 and ***p < 0.001. **(D)** Principal coordinates analysis (PCoA) with Bray-Curtis distance (β-diversity) based on gut metagenomic species profiles, PERMANOVA, *R*^2^ = 0.469, *p* = 0.002.

Metagenomic sequencing generated an average of 1.51 billion raw reads comprising 14.3 billion bases from 16 fecal samples (8 of SBS rats and 8 of Sham rats). After quality-filtering and removal of host genome, we obtained an average of 65,548,858 optimized reads per sample. The Venn diagram presented the distribution of bacterial species, with 76.5% (15,272 in 19,961) shared by the two groups and 798 unique to the SBS group (Figure 1A). Compared with the Sham group, significantly decreased microbial richness and α-diversity was found in SBS group (Chao1, *p* = 0.0003; Shannon, *p* = 0.0047, Figures 1B, C). PCoA analysis showed that the intergroup plots were well-separated and clustered into two groups (PERMANOVA: *R*^2^ = 0.469, *p* = 0.002, Figure 1D).

### Changes in SBS microbiome community composition

**FIGURE 2:**
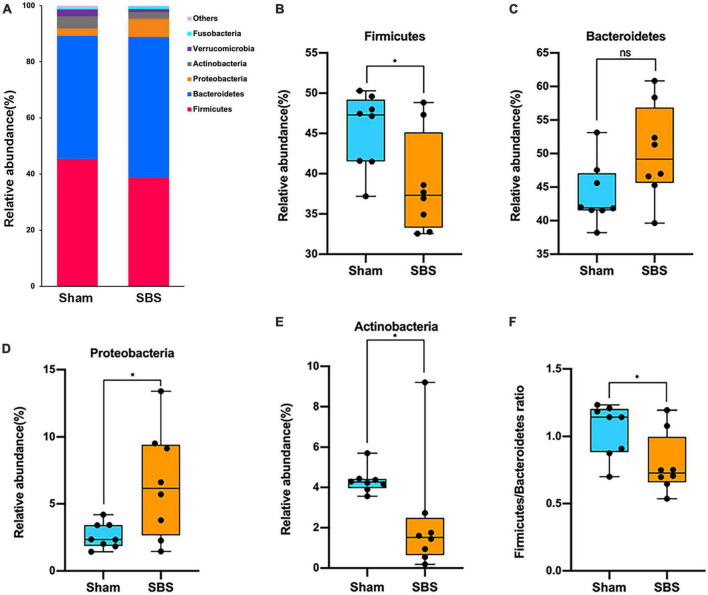
Phylum-level changes in gut microbiome community composition. **(A)** The relative frequency of top abundant taxa in short bowel syndrome (SBS) and Sham groups at the phylum level. **(B–E)** Comparison of the relative abundance of Firmicutes **(B)**, Bacteroidetes **(C)**, Proteobacteria **(D)**, and Actinobacteria **(E)** between SBS, and Sham groups. **(F)** The values of Firmicutes/Bacteroidetes of SBS were significantly lower than the Sham group. The dot represents one value from individual participants. The box plots represent median and interquartile range (IQR), and the whiskers indicate the 10th and 90th percentiles. *p < 0.05, ns, no significance.

At the phylum level, the 6 most abundant phyla identified in both groups were Firmicutes, Bacteroidetes, Proteobacteria, Actinobacteria, Verrucomicrobia, and Fusobacteria, totally accounting for over 99% of the bacteria (Figure 2A). However, the relative abundance of each phylum in SBS was remarkably different when compared to the Sham group. The rats in SBS group harbored dramatically greater proportion of Proteobacteria (*p* = 0.049, Figure 2D), while the proportions of Firmicutes (*p* = 0.038, Figure 2B), and Actinobacteria (*p* = 0.010, Figure 2E) significantly decreased. No statistical significance of the relative abundance of Bacteroidetes was detected between groups (*p* = 0.105, Figure 2C). We also observed significantly lower Firmicutes/Bacteroidetes (F/B) ratio in SBS group (*p* = 0.038, Figure 2F).

**FIGURE 3:**
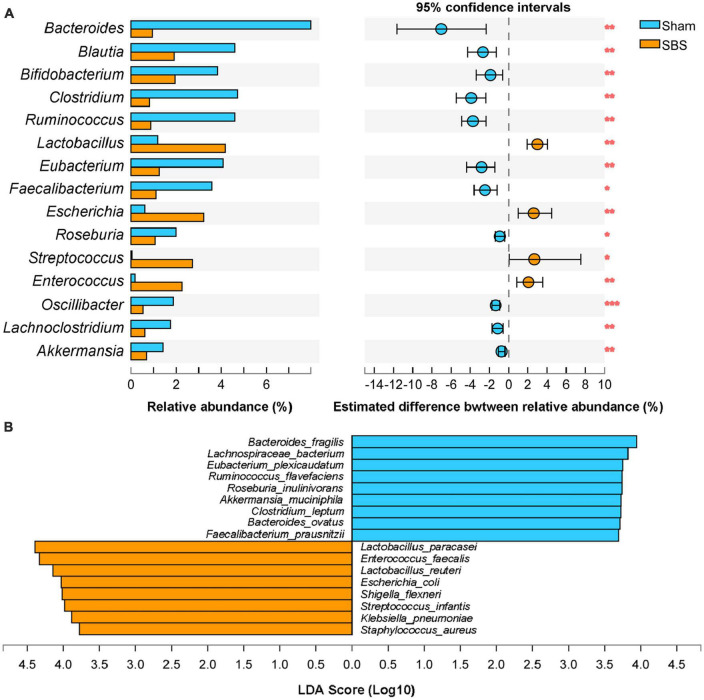
Genus and species-level discrimination between short bowel syndrome (SBS) and Sham rats. **(A)** Bar plot showing the top 15 bacterial genera with statistical difference in the relative abundance between SBS and Sham groups. The right graph exhibited the estimated difference of each genus between two groups with 95% confidence intervals (CI) calculated by Hodges-Lehmann method. Mann–Whitney test, *p < 0.05, **p < 0.01, and ***p < 0.001. **(B)** Linear discriminant analysis (LDA) effect size (LEfSe) analysis of enriched fecal microbial species in SBS and Sham groups (LDA > 3.5, *p* < 0.05).

The top 15 discriminative genera identified between two groups were presented in Figure 3A. The microbiome of SBS rats was dominated by *Lactobacillus*, meanwhile, *Escherichia* and *Enterococcus* were also markedly enriched in SBS. Another unique feature of SBS rats was the elevated representation of *Streptococcus*. Conversely, a variety of commensal bacterial genera including *Bacteroides*, *Blautia*, *Bifidobacterium*, *Clostridium*, *Ruminococcus*, *Eubacterium*, *Faecalibacterium*, *Roseburia*, *Oscillibacter*, *Lachnoclostridium*, and *Akkermansia*, were significantly abundant in Sham rats but depleted in SBS rats.

Effect size approach was applied to further explore specific taxa differentially enriched or depleted in SBS that make them to be potential biomarkers. In total, 17 species were identified with LDA > 3.5 (Figure 3B). SBS rats were characterized by 8 species, including *Lactobacillus paracasei*, *Enterococcus faecalis*, *Lactobacillus reuteri*, *Escherichia coli*, *Shigella flexneri*, *Streptococcus infantis*, *Klebsiella pneumonia*, and *Staphylococcus aureus*; while the distinctive markers of Sham rats were *Bacteroides fragilis*, *Lachnospiraceae bacterium*, *Eubacterium plexicaudatum*, *Ruminococcus flavefaciens*, *Roseburia inulinivorans*, *Akkermansia muciniphila*, *Clostridium leptum*, *Bacteroides ovatus, and Faecalibacterium prausnitzii*.

As we observed heterogeneity in the body weight evolution of SBS rats, we looked for its relationships with their predominant fecal microbes. At postoperative day 28, the relative abundance of *Lactobacillus* was positively correlated with the body weight of SBS rats (*r* = 0.81, *p* = 0.02, Supplementary Figure 2A), while the proportion of Proteobacteria was negatively correlated with the body weight (*r* = −0.74, *p* = 0.046, Supplementary Figure 2B).

### Functional characterization of the SBS microbiome

**FIGURE 4:**
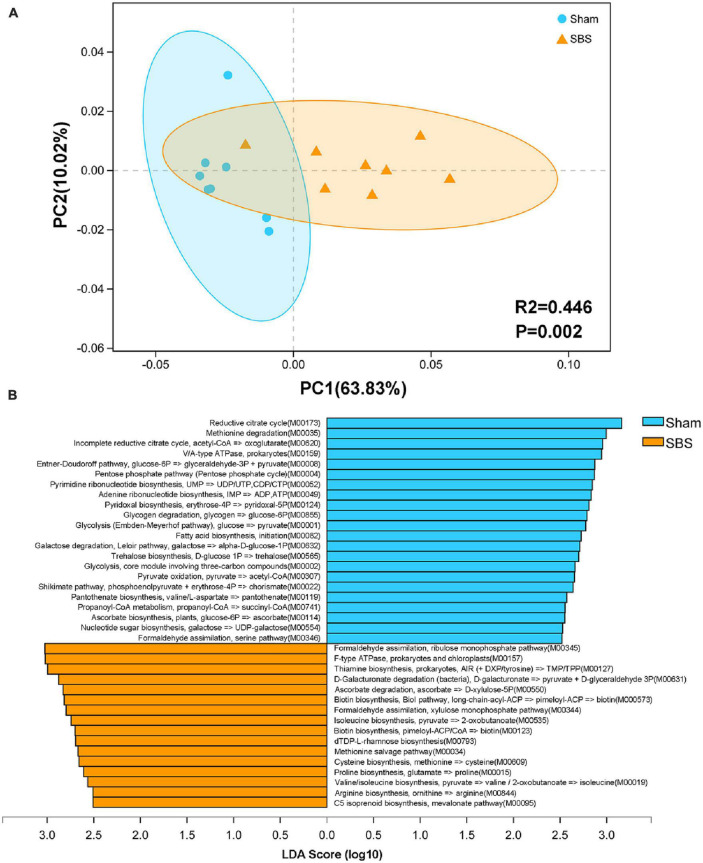
Functional characterization of the short bowel syndrome (SBS) microbiome. **(A)** Principal coordinates analysis (PCoA) of Bray-Curtis dissimilarity between Sham and SBS rat models based on the relative abundance of level 3 Kyoto Encyclopedia of Genes and Genomes (KEGG) pathways, PERMANOVA: *R*^2^ = 0.446, *p* = 0.002. **(B)** Linear discriminant analysis (LDA) effect size (LEfSe) analysis of KEGG modules that were differentially abundant in SBS and Sham groups (LDA > 2.5, *p* < 0.05).

To further provide a functional profiling of the microbial communities, we annotated the ORFs to KEGG pathways and modules. Despite of some overlaps, the PCoA score plot showed distinct separation of the two groups based on level 3 KEGG pathways (PERMANOVA: *R*^2^ = 0.446, *p* = 0.002; Figure 4A). Additionally, LEfSe analysis identified 38 KEGG modules which were differentially abundant between SBS and Sham groups (LDA > 2.5, *p* < 0.05; Figure 4B). Among them, pathways accounting for carbohydrate metabolism and ribonucleotide biosynthesis were particularly enriched in Sham group, whereas modules involved in vitamin metabolism and amino acids biosynthesis were dominant in SBS group.

### Differential metabolic patterns perturbed in SBS

**FIGURE 5:**
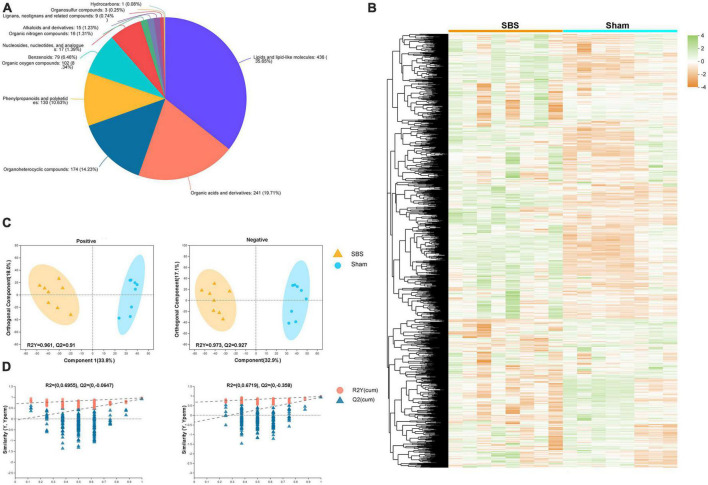
Metabolome signatures of fecal samples from short bowel syndrome (SBS) and Sham rats. **(A)** Pie chart illustrating the abundance ratio of different classes of fecal metabolites based on human metabolome database (HMDB). **(B)** Fecal metabolites of SBS and Sham groups detected by metabolomic profiling were normalized, centered, clustered, and presented by heatmap. **(C)** Orthogonal partial least squares discriminant analysis (OPLS-DA) of positive and negative ions detected in fecal samples from SBS and Sham rat models. **(D)** The response permutation testing of OPLS–DA for positive and negative ions.

We performed untargeted LC-MS analysis to gain a deeper understanding of the fecal metabolome signatures of SBS and Sham rats. In total, 1,577 metabolites with known chemical identity were detected from all samples, including 892 in the positive and 685 in the negative ion mode. Cluster analysis of these metabolites was visualized as a heatmap in Figure 5B. Each metabolite was then assigned to chemical superclass based on Human Metabolome Database (HMDB). We found that most metabolites belonged to “lipid and lipoid-like molecules,” “organic acids and derivatives,” “organoheterocyclic compounds,” and “phenylpropanoids and polyketides,” the total proportions of which were over 80% (Figure 5A). To maximize the dissociation between two groups, a supervised method of pattern recognition, OPLS-DA, was used to evaluate the quantitative variation of metabolites. The OPLS-DA model exhibited clear separation and significant discrimination of the fecal metabolites between SBS and Sham group in both ion modes (Figure 5C), and the permutation tests (*n* = 200) indicated good validity and satisfactory prediction capabilities of the model (Figure 5D).

**FIGURE 6:**
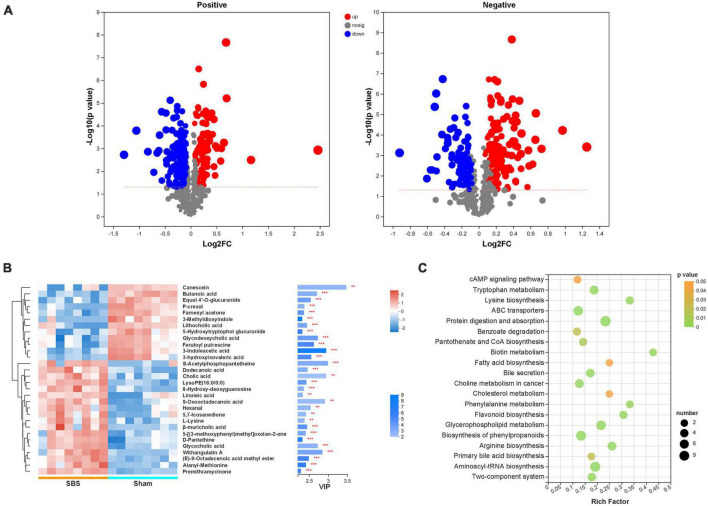
Discrepant metabolic patterns in short bowel syndrome (SBS) and Sham rats. **(A)** Volcano plots of the differential fecal metabolites with variable importance for the projection (VIP) > 1 and *P* < 0.05 between SBS and Sham groups. Each symbol represents the identified metabolite, red represents significantly up-regulated metabolites, blue represents significantly down-regulated metabolites, and gray represents the metabolites those did not differ. **(B)** The hierarchical cluster and heatmap showing the top 30 significantly altered metabolites in each sample (VIP > 2, *P* < 0.05). Red and blue colors represent high and low levels of metabolites, respectively. The bar graph shows the VIP scores of each metabolite. *p < 0.05, **p < 0.01, and ***p < 0.001. **(C)** Bubble diagrams showing the top 20 enriched Kyoto Encyclopedia of Genes and Genomes (KEGG) pathways of the differential metabolites. Abscissa variations indicate the degree of enrichment (Rich factor), and the vertical axis shows the KEGG pathway information. The color scale indicates significance levels, and the node size represents the number of discriminative metabolites in the mapping pathway.

Significantly differential metabolites defined as OPLS-DA VIP > 1 and *P* < 0.05 were selected, and the volcano plots of metabolites were present in Figure 6A. Compared to the Sham group, 184 positive-ion and 92 negative-ion metabolites were down-regulated in SBS group, and 90 positive-ion and 134 negative-ion metabolites were up-regulated in SBS group. To further identify metabolic biomarkers for SBS, the top 30 distinguished metabolites (VIP > 2 and *P* < 0.05) were clustered (Figure 6B). The dramatically elevated primary BAs, including cholic acid (CA), β-muricholic acid (βMCA) and glycocholic acid (gCA), were specific for SBS. In contrast, two secondary BAs, lithocholic acid (LCA) and glycodeoxycholic acid (gDCA), were dominant in fecal samples from Sham group. The feces of Sham rats were also enriched with two SCFAs (butanoic acid and 3-hydroxyisovaleric acid) and products of amino acid metabolism (3-indoleacetic acid, 3-methyldioxyindole and p-cresol). Several other fatty acids, such as dodecanoic acid and linoleic acid, and hexanal belonging to medium-chain aldehyde were distinctively up-regulated in SBS feces. Enrichment analysis based on KEGG annotation was performed to highlight the differential metabolic pathways between Sham and SBS rats. Consistent with the different metabolite abundances observed, the pathways involved in tryptophan metabolism, fatty acid biosynthesis, biliary secretion, cholesterol metabolism, phenylalanine metabolism, and primary BA biosynthesis were significantly different between the two groups (Figure 6C).

### Integrated correlation analysis of gut microbial species and fecal metabolites

**FIGURE 7:**
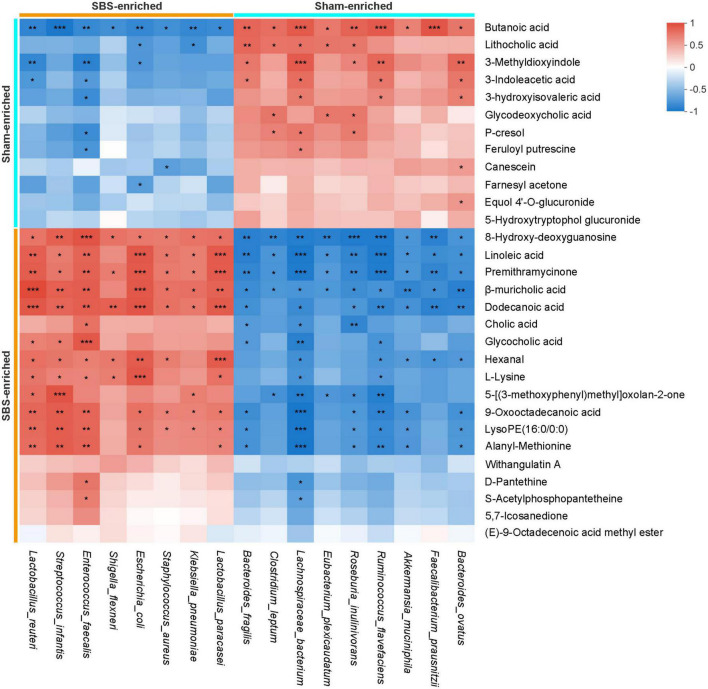
Integrated correlation of gut microbial species and fecal metabolites. Heatmap of Spearman’s rank correlation coefficients between differentially abundant species and metabolites from short bowel syndrome (SBS) and Sham rats. The red or blue color in each square represents positive or negative relationship, respectively. Statistically significant correlations were marked with asterisks (*p < 0.05, **p < 0.01, and ***p < 0.001).

The Spearman’s rank correlations were calculated to explore the potential functional relationships between the 17 distinctive microbial species and top 30 differential metabolites, and a total of 215 significant correlations were revealed (Figure 7). Briefly, metabolites and microbes simultaneously enriched in SBS or Sham group were positively associated. In particular, a number of positive correlations of beneficial microorganisms, such as *Lachnospiraceae bacterium* and *Roseburia inulinivorans*, with SCFAs and secondary BAs were uncovered, suggesting the possible metabolic interactions between each other. Meanwhile, the negative relationships between Sham-enriched microbes and SBS-enriched metabolites were also observed, which reflected the impact of depletion of normal resident bacteria on metabolic patterns in SBS.

## Discussion

In the present study, through the combination of metagenomic sequencing and untargeted metabolomics techniques, we investigated the signatures and correlations of differential bacterial species and fecal metabolites in the gut ecosystem of SBS rats. As gut microbiota contributes to intestinal adaptation either by itself or by producing host-interactive metabolites (Marchix et al., 2018), our findings may primarily provide evidence for a stool-based approach for gut adaptation assessment as well as lay the foundation for developing new strategies to facilitate intestinal adaptation in SBS patients.

Dysbiotic gut microbiome in SBS has been reported by several literatures, which largely employed 16S rRNA gene sequencing (Neelis et al., 2019). In agreement with those studies, we observed decreased species-level diversity and richness as well as significantly altered microbiome community structure in SBS compared to Sham rats. However, no change in the microbial α-diversity was observed in a preclinical mouse model of SBS with 50% of the proximal small bowel resected (Sommovilla et al., 2015). This may partly attribute to the shorter length of resected intestine and the preservation of ileocecal valve. Considering that the type II SBS with extensive ileocecal resection and jejuno-colonic anastomosis is more commonly observed in SBS patients and usually shows severe clinical manifestation and poor prognosis (Pironi et al., 2018; Tappenden, 2023), our rat SBS models allow to study the pathological conditions of the majority of SBS patients. Of note, we have previously demonstrated that SBS rats with ileocecal resection exhibit a more severe gut dysbiosis (Huang et al., 2020). However, owing to the different full length of small bowel, our 75% resection could lead to different length of the remaining small intestine, which may induce potential variability in the intestinal adaptation and the fecal microbial community among SBS rats.

Regarding the compositional alterations, the gut microbiota in rats with SBS had lower Firmicutes and Actinobacteria but greater abundance of Proteobacteria, primarily the genus *Escherichia*, relative to Sham controls. The overrepresentation of Proteobacteria has been reported in numerous studies concerning SBS and intestinal failure (Neelis et al., 2019), their pathogenic potential and proinflammatory effect contribute to central line-associated bloodstream infection (CLABSI) and intestinal failure-associated liver disease (IFALD) in SBS patients (Wang et al., 2017). However, the decreased Firmicutes in SBS rats was unexpected because many studies have reported dominance of Firmicutes in SBS feces (Budinska et al., 2020; Boutte et al., 2022; Fourati et al., 2023), of which, the highly abundant *Lactobacillus* was the major contributor. Despite the similar increasing of *Lactobacillus* in our study, we further identified 8 commensal genera within Firmicutes that were strikingly depleted in SBS but abundant in Sham rats. This modification was consistent to a prior study (Piper et al., 2017) and partially explained the Firmicutes-deficient gut microbiome and lower F/B ratio in SBS. Besides, we collected fecal samples 28 days after operation while a recent study analyzed the gut microbiome of SBS rats at postoperative day 15 (Fourati et al., 2023), the different time points may also lead to the contradiction. Interestingly, *Streptococcus*, the oral-associated bacteria, significantly increased in fecal samples from SBS rats, which was also observed in patients with inflammatory bowel disease showing genetically related oral and intestinal streptococcal strains isolated from the same patient (Abdelbary et al., 2022). Our finding was concordant with previous studies concerning pediatric and adult SBS patients (Davidovics et al., 2016; Budinska et al., 2020; Thänert et al., 2021) and suggested ectopic gut colonization by oral bacteria in SBS.

Our LEfSe analysis identified 17 bacterial species linked with SBS, which was a prerequisite for investigating the biomarkers for clinical applications. We noted that the down-regulated species characterizing rats with SBS mainly belonged to commensal genera within the phylum Firmicutes, such as *Clostridium*, *Ruminococcus*, and *Lachnoclostridium*, which are capable of fermentation of carbohydrate and production of SCFAs (Tanes et al., 2021). Thus, deficiency of these beneficial species indicated a lower energy-harvest capacity, and partly contributed to the poor gain weight of SBS rats. Two species, *Bacteroides fragilis* and *Bacteroides ovatus*, were also detected with a reduction in rats with SBS. As a part of the commensal gut flora, the benefits of *Bacteroides fragilis*, including preventing pathogens colonization and translocation, restoring gut barrier integrity and maintaining microbial balance, have been determined in mounting studies (Deng et al., 2018; Gautier et al., 2022). Furthermore, our analysis showed enrichment of *Akkermansia muciniphila* in Sham rats, which was proposed to be a contributor to metabolic heath and intestinal adaptive immune responses (Dao et al., 2016; Ansaldo et al., 2019). Therefore, we speculated that decreased *Akkermansia muciniphila* in SBS may confer a risk for adverse metabolic effects and disturbed host immune function. Surprisingly, *Lactobacillus paracasei* and *Lactobacillus reuteri* unexpectedly thrived in fecal samples of SBS rats. Since *Lactobacillus* species are more commonly found in small intestine (Frank et al., 2007), their dominance in the feces of SBS rats may result from the dramatically altered gut environment caused by massive ileocecal resection, which probably favors the growth of *Lactobacillus* or prevents the implantation of other bacteria. Given that *Lactobacillus paracasei* and *Lactobacillus reuteri* are regarded as probiotics and lack of *Lactobacillus* species was associated with poor growth in pediatric SBS patients (Sikorska and Smoragiewicz, 2013; Piper et al., 2017), the *Lactobacillus*-prevalent microbiome in SBS may reflected a better intestinal adaptation. Additionally, a positive correlation between postoperative food intake and fecal *Lactobacillaceae* abundance has also been demonstrated in SBS rats (Fourati et al., 2023).

Metagenome-based analysis allows us to gain deep insights into the metabolic functions of the intestinal microbiota. Our current study showed that gut microbiome of SBS rats was deficient with carbohydrate metabolism associated KEGG modules, such as glycolysis and pentose phosphate pathway. Such reduction was mostly attributed to the shifted proportion of bacterial species, especially those from phylum Firmicutes. Besides, the low capacity for energy harvest of intestinal microbiome strongly signified malnutrition status and poor growth in SBS subjects. The modules related to branched-chain amino acids (valine and isoleucine) biosynthesis were enriched in SBS rats, which was consistent with a previous study and was regarded as a metabolic feature of poor-growth SBS children (Piper et al., 2017).

Gut metabolites are key mediators of host-microbiota interactions and can be significantly shaped by gut microbiome. We developed reliable and robust OPLS-DA models revealing remarkably distinct metabolic profiles in SBS relative to Sham rats. Among the altered metabolites, we clarified two SCFAs, butanoic acid and 3-hydroxyisovaleric acid, that were severely reduced in feces of SBS rats. SCFAs, particularly butyrate, are known to serve as energy-supplying fuel for intestinal epithelial cell (Schönfeld and Wojtczak, 2016), maintain epithelial homeostasis via modulating the production of IL-18 and regulate immune response by enhancing the function of colonic regulatory T cells (Smith et al., 2013; Macia et al., 2015). Moreover, previous studies reported that supplementation of SCFAs, either by dietary or intravenous, improved adaptation of both small bowel and colon in SBS animals (Marchix et al., 2018). Our correlation analysis further confirmed the strong positive correlations between SCFAs and species from *Lachnospiraceae* and *Ruminococcaceae* families, which are the most important SCFAs producers and also decreased in SBS as expected. Nevertheless, the exact SCFA production could not be simply determined by fecal concentrations because of the absorption by colonic cells (Atia et al., 2011). Furthermore, the decreased fecal SCFAs concentrations may also result from the dilution caused by increased fecal water loss in SBS rats. Yet, given that the fecal SCFAs reductions were concomitant with a decrease of SCFA-produce species in SBS, our results may still suggest a reduced production of SCFAs by gut microbiota. The shortage of SCFAs makes them, and the bacteria that produce them, potential therapeutic agents to facilitate intestinal adaptation in patients with SBS.

We revealed a dysmetabolism of BAs in SBS rats featured by a significant elevation of fecal primary BAs and a reduction in fecal secondary BAs, which agreed with our metagenomic analysis demonstrating decreased relative abundance of species within genera *Bacteroides*, *Eubacterium*, and *Clostridium* known to possess bile salt hydrolase (BSH) activity and/or 7α-dehydroxylating ability. The altered fecal BA patterns were also observed in a piglet SBS model and human SBS patients (Pereira-Fantini et al., 2014; Kastl et al., 2022). As the physiological concentration of secondary BAs is crucial for exerting anti-inflammatory actions in intestine (Ward et al., 2017), the failure of transformation of primary to secondary BAs due to gut dysbiosis may contribute to the pro-inflammatory status in SBS. Moreover, considering that LCA and DCA are more potent activators of farnesoid X receptor (FXR) than CA, lack of secondary BAs in SBS may lead to impaired activation of intestinal FXR and subsequent reduction in plasma fibroblast growth factor 19 (FGF19) levels, a mediator of FXR-dependent suppression of hepatic bile acid synthesis (Pereira-Fantini et al., 2014; Kliewer and Mangelsdorf, 2015; Kastl et al., 2022). The attenuated feedback of FXR-FGF19 axis probably causes cholestasis and even IFALD, a major cause of death in SBS patients.

The KEGG enrichment analysis of the differential metabolites exhibited disturbed tryptophan metabolism, accordingly, we found decreased levels of indole derivatives, such as 3-indoleacetic acid (IAA), in SBS feces. Indole and its derivatives are products of microbial tryptophan metabolism and many of them act as endogenous ligands for aryl hydrocarbon receptor (AhR) (Hubbard et al., 2015). The main bacterial species capable of generating IAA include *Bacteroides ovatus*, *Bacteroides fragilis*, and *Clostridium spp.* (Lamas et al., 2016; Dodd et al., 2017), which were also confirmed by our microbes-metabolites correlation analysis. Activation of AhR drives the expression of interleukin-10 (IL-10) receptor on intestinal epithelial cells, which in turn promote barrier function and epithelial wound healing (Lanis et al., 2017). In addition, indole derivatives can protect intestine against pathogenic infection and limit mucosal inflammation by stimulating IL-22 expression via activating AhR (Lee et al., 2011; Monteleone et al., 2011). As a result, we speculated that the lack of indole derivatives may be one of the possible factors contributing to damaged intestinal permeability and enhanced gut inflammation in SBS.

Our results are limited by several factors, the most important being the absence of data directly reflecting the intestinal adaptation in SBS. Although we recorded the body weight evolution of SBS rats, parameters such as food intake, intestinal length, villus height and crypt depth are needed in further studies. Another limitation is the small sample size and more samples may reduce the bias resulting from heterogeneity of gut microbiome between individuals. In addition, given that our rat models cannot completely recapitulate the characteristics of SBS patients, larger cohorts of patients should be investigated in clinical studies. Moreover, we lacked the cultivation-based confirmation of the results from metagenomic sequencing, as combining molecular and culture-based approaches may make the results more convincing.

In summary, we demonstrated a dysbiotic gut environment in rat SBS models, characterized by depleted commensal microbial species, increased opportunistic pathogens, and disrupted SCFAs, BAs and indole metabolism. These microbiome and metabolome alterations may contribute to the proinflammation status of SBS and delay gut adaptation, which in turn provide promising targets for therapies.

## Data availability statement

The data presented in the study are deposited in the National Center for Biotechnology Information (NCBI) repository (https://www.ncbi.nlm.nih.gov/), accession number: PRJNA936446.

## Ethics statement

This animal study was reviewed and approved by the Animal Experimental Ethics Committee of the Shanghai Ninth People’s Hospital, Shanghai Jiao Tong University School of Medicine.

## Author contributions

YL and FG designed the study. YH, JJ, and DY performed the research. YH, JJ, and FG analyzed the data. YH, JJ, DY, FG, and YL wrote the manuscript. All authors contributed to the article and approved the submitted version.
