# Supplementary material for: Altered fecal microbiome and metabolome profiles in rat models of short bowel syndrome
Source: Front Microbiol. 2023 Jun 9;14:1185463. doi: 10.3389/fmicb.2023.1185463 (PMC10289890; doi:10.3389/fmicb.2023.1185463)
Supplement: Supplementary file 1 [file Data_Sheet_1.PDF]

Supplementary materials

Figure S1 Postoperative body weight follow-up of the rats in short bowel syndrome (SBS) and Sham groups. Body weight was presented as percentage of the preoperative weight. (A) Body weight evolution of the SBS and Sham rats during 28 days after surgery. Data are shown as mean ± SD. (B) Body weight of SBS and Sham rats at postoperative day 28. The dot represents one value from individual participants. The box plots represent median and interquartile range (IQR), and the whiskers indicate the 10th and 90th percentiles. Mann-Whitney test, $^{***}P < 0.001$, $^{****}P < 0.0001$.

Figure S2 Correlations between body weight at postoperative day 28 and relative abundance of fecal *Lactobacillus* (A) and Proteobacteria (B) in rats with short bowel syndrome (SBS), analyzed by Spearman rank correlation. Body weight was presented as percentage of the preoperative weight.

Figure S1

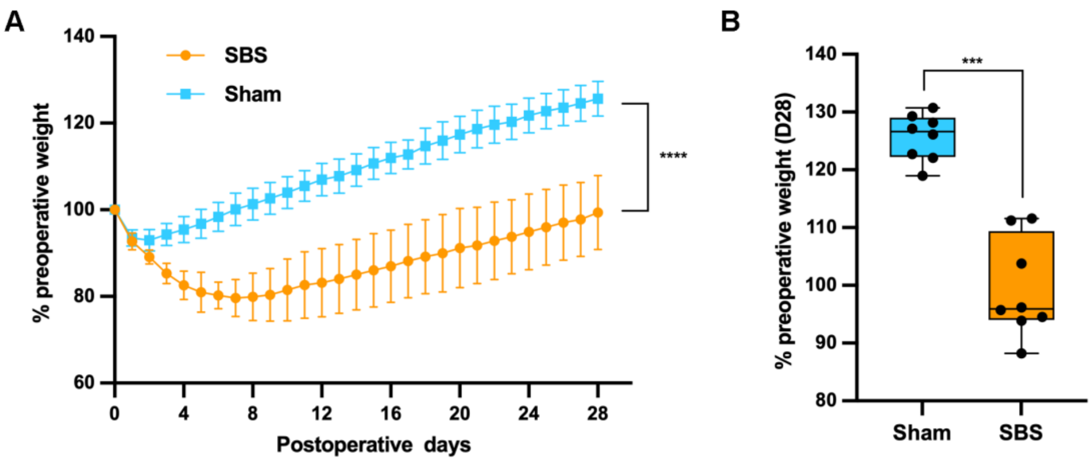

Figure S1 Postoperative body weight follow-up of the rats in SBS and Sham groups.

Figure S2

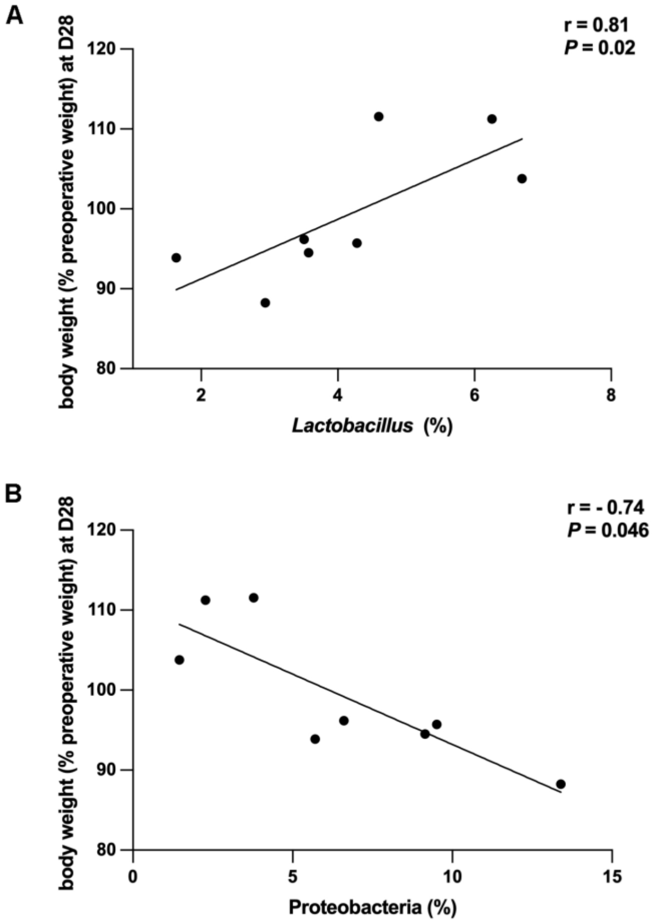

Figure S2 Correlations between body weight at postoperative day 28 and relative abundance of *Lactobacillus* and Proteobacteria in rats with SBS.
